# Constitution-specific features of perspiration and skin visco-elasticity in SCM

**DOI:** 10.1186/1472-6882-14-24

**Published:** 2014-01-15

**Authors:** Young-Min Kim, Boncho Ku, Chang Jin Jung, Jaeuk U Kim, Young Ju Jeon, Keun Ho Kim, Jong Yeol Kim

**Affiliations:** 1 Medical Research Division, Korea Institute of Oriental Medicine, Daejeon 305-811, Republic of Korea

**Keywords:** Sasang constitutional medicine, Skin, Visco-elasticity, Elasticity hysteresis, Perspiration

## Abstract

**Background:**

Human skin properties have been used as an important diagnostic component in traditional medicine as they change with health conditions. Sasang constitutional medicine (SCM) puts emphasis on the recognition of the constitution-specific skin features prior to the diagnostic decision of health. In this work, in search of skin-characteristics effectively reflecting SCM features, we compared several skin properties such as perspiration, visco-elasticity, elasticity, and elasticity hysteresis, in several candidate body parts.

**Methods:**

We conducted a clinical study in which a total of 111 healthy females aged 50 – 70 years participated with their Sasang constitution (SC) types determined objectively by the Sasang constitutional analytic tool. Perspiration on the skin surface was estimated by using a capacitance sensor to measure the amount of moisture on the palm, forehead, and philtrum before and after a heating stimulus. We acquired the visco-elasticity, elasticity, and elasticity hysteresis at the forearm by Dermalab’s elasticity sensing device. An analysis of covariance (ANCOVA) was conducted to evaluate the effect of SC on the nine skin features acquired.

**Results:**

The visco-elasticity of the forearm of the Soeum-in (SE) group was significantly lower than that of the Taeeum-in (TE) group (F = 68.867, p < 0.001), whereas the elasticity hysteresis of the SE group was higher than that of the TE group (F = 10.364, p < 0.01). The TE group had more perspiration on the forehead than the SE group (F = 9.050, p < 0.01). The SE group had a large perspiration difference between the philtrum and the forehead compared with the TE group (F = 7.892, p < 0.01).

**Conclusions:**

We found four significant skin features that reflect the inherent constitutional attributes of the TE and SE groups in accordance with SCM literature; the visco-elasticity, elasticity hysteresis, perspiration on the forehead and philtrum. Our findings are based on a novel interpretation of the SCM literature and will contribute to developing the constitutional health status evaluation system in SCM.

## Background

In recent years, Sasang constitution (SC) medicine has attracted widespread interest from fields such as integrative medicine and personalized medicine because it offers important benefits regarding the inherent categorization of human types for the diagnosis of one’s holistic health status [1,2]. SC is a unique form of traditional Korean medicine (TKM) that divides people into four constitutional types (Taeyang-in: TY, Taeeum-in: TE, Soyang-in: SY, and Soeum-in: SE), which differ in inherent mind-body characteristics such as external appearance, personality traits, susceptibility to particular diseases, drug responses, and balance of internal organ functioning [3]. In contrast with the diagnostic procedure of traditional Chinese medicine, which places the most importance on the 'syndrome,’ SC medicine places its emphasis on the inherent 'constitution’, and the therapeutic decision is thus primarily based on the individual’s SC type [4].

One way to describe SC in TKM is to observe the physical properties of the human skin by palpation [5]. In particular, the distinct mechanical and perspiration differences between TE and SE have been established in important TKM literature. In Donguisusebowon [6] and Sasangyolam [7], traditional Korean doctors have summarized the mechanical characteristics of the two SC types as follows:

TE inherently has solid, stiff, and thick skin, whereas SE has buoyant and soft skin.

Moreover, in Donguisusebowon [6,8], the different sweating properties of TE and SE have been used to assess how much one’s health status has improved:

When health status of TE gets better, he/she perspires at the variety of body sites such as head, eyebrows around, lip, and chest etc. On the other hand, SE perspires at only philtrum around and the amount of the sweat is much less compared to that of TE.

Using these traditional empirical reports as a basis for study, Lee et al. qualitatively analyzed the relevance of the physical properties of the human skin to SC by conducting clinical studies with 1,079 subjects who were registered in a SC information bank. Lee et al. confirmed that the physical properties of skin could be useful indicators for SC diagnosis [9,10].

However, there have been few studies on the quantitative relevance of SC and the skin properties involved in determining constitution type. Kang *et al.*[11], Song *et al.*[12] and Lee *et al.*[9] reported that skin elasticity and thickness on the palm and dorsal sides of the hands of TEs and SEs are significantly different. Kim *et al.*[13] found that the roughness on the surface of the skin of TEs was higher than that of SYs. These pilot studies on the mechanical features of human skin did not contain a sufficient amount of clinical data to show that their results were valid. Jung *et al.*[14] investigated different humidity characteristics as described by SC and concluded that TEs have a higher humidity overall than SEs. The two groups did not show different characteristics at different body sites.

In this work, we refine the definition of the measurable quantities and measuring locations, and reexamine the constitution-specific skin properties. For this purpose, firstly we introduce three skin elasticity-affected quantities such as elasticity, visco-elasticity, and elasticity hysteresis. Second, we select an easily accessible and repeatability-guaranteed body parts for the measurement; we choose philtrum, forehead and palm for the perspiration measurement, and choose forearm to measure the skin elasticity-affected quantities. As a result, we will show some novel skin features which are effective in distinguishing the SC types.

## Methods

### SC diagnosis and subjects

Several recent studies showed attempts to develop SC diagnostic systems based on objective and quantitative measurements of various human traits. For instance, Do *et al.*[15-17] succeeded in extracting the geometrical features from frontal and lateral facial images of the four SC types. Kim *et al.*[18,19] attempted to classify the SC types by using vocal features. Moreover, full-body information such as weight, body mass index, and the ratios of chest, hip and head circumferences have been investigated by Jang *et al.*[20-22] to objectively verify SC relevance.

Based on the above studies, the Sasang constitutional analytical tool (SCAT) was developed by the Korea Institute of Oriental Medicine to measure these characteristics [22,23]. It can provide Korean medical doctors (KMDs) with information for the treatment of various diseases by using integrated SC scores. The most updated classification accuracy of the SCAT with respect to the two SC groups (TE and SE) was reported to be 87.1% and 90.1% in the male and female subjects groups, respectively [23]. The high accuracy could be reached with exclusion of some low constitution scored subjects; specifically, we applied a cut-off value for the integrated SC score, 1.6, with which it guaranteed the accuracy increase by 14.7% and 4.6% in male and female sub-groups compared to the entire subject group [23].

The subjects consisted of people classified into the typical TE or SE categories with high SCAT classification scores (a cut-off value of the integrated SC score >1.6). The diagnosis results were confirmed by the independent assessment of two KMDs who had at least five years of clinical experience in Sasang constitutional medicine. TEs and SEs were classified by constitution into groups of 65 and 46, respectively.

Two KMDs with at least five years of clinical experience participated to diagnose the subjects’ health states. To determine a subject’s health state, doctors used the four methods of diagnosis (observation, auscultation and olfaction, inquiry, and pulse feeling and palpation). For the quantitative health evaluation, doctors used a visual analogue scale (VAS) scoring with scores ranging from 0 (seriously ill) to 100 (complete physical and mental well-being). For the calibration of the participating doctors’ VAS scores, a reference score of 40 was used to separate healthy from diseased subjects [24]. Subjects who were diagnosed with light common aged-related symptoms such as borderline hypertension which could be controlled by daily preventive self-care (and minimal level of medication) were categorized into the healthy group, and more severe symptoms which required some medication were categorized into the diseased group. The constitutional health score was then determined by averaging the VAS scores assigned by the two doctors. To guarantee the consistency of the diagnosis, the subjects whose VAS differences between the two KMDs were more than 20 were excluded from the study.

A total of 111 healthy women aged 50–70 years finally participated in this experiment. The subjects used no cosmetics before the measurements and were acclimatized for a minimum of 20 min in controlled conditions to prevent temperature and humidity variations from affecting the acquisition of skin moisture data. The controlled conditions were empirically obtained by the pilot study in which we found appropriate temperature and humidity values (27°C and 35%) to induce moderate perspiration from the heating stimulus in the back of subjects for ten minutes. The room conditions were measured before and after the heating stimulus experiments and were controlled in the ranges of 27.0±0.52°C temperature and 37.2±4.3% humidity. Before the heating stimulus, visco-elasticity was measured three times at the center of the left volar forearm. The moisture level at the palm, forehead, and philtrum was measured twice before and twice after the heating stimulus. Subjects who had no change in moisture level at any of the measuring sites were excluded from the experiment. Approval of the clinical trials was obtained from the institutional review board at the oriental hospital of Daejeon University, Korea (IRB No, M2012-01) and written informed consent to take part was obtained from all participants in this study.

### Skin elasticity measurement

Several objective methods to measure the elasticity of human skin have been studied by cosmetic, rehabilitation, surgical research fields, and others. Suction chamber devices were commonly used to non-invasively determine the visco-elastic properties of the skin [25,26]. In this study, The Dermaflex device (CORTEX TECHNOLOGY, Denmark) with a high sensing repeatability [27] was used to measure the visco-elasticity coefficients, as well as the elasticity coefficients.

The elasticity coefficient (*E*) of the skin surface was estimated by measuring the suction pressure (*p*) when the skin was stretched at a specified location. The strain deviation (∆*x*) and suction area, which consist of the radius (*r*) and the thickness (*s*) of the skin, can be set to constant values. Therefore, the *E* value of the skin surface is obtained by

(1) $$E=\varphi \cdot p\cdot \frac{r^{4}}{\mathrm{\Delta x}\cdot s^{3}}\;$$

where ∆*x* is strain deviation, *φ* is a constant value, *p* is surface pressure, *r* is the radius of the surface, and *s* is the thickness of the surface.

Visco-elasticity (*V*_*E*_) was defined by the ratio of the elasticity coefficient (*E*) to recovery time (*T*_*r*_) in the following Equation (2). *T*_*r*_ represents the time required for the skin to recover from the maximally stretched position to the initial position.

(2) $$V_{E}=\frac{E}{T_{r}/260}\;$$

However, when elasticity measurements are performed consecutively in the same location, the elasticity of the skin gradually decreases. This hysteresis of elasticity can be used to evaluate the degree of skin fatigue. In our study, the elasticity hysteresis (*E*_*HYS*_) was defined as the difference between the first elasticity coefficient value (*E*_1_) and the third elasticity coefficient value (*E*_3_) using

(3) $$E_{\mathrm{HYS}}=\frac{E_{1}-E_{3}}{E_{1}}\;$$

where *E*_i_ is the i^th^ elasticity coefficient.

The above three features (E, V_E_, and E_HYS_) were measured on the left volar forearm three times. Because the accuracy of the elasticity measurements would have been influenced by underlying muscle movements, we controlled for this by keeping the angle of the elbow and wrist in a relaxed state at 90 and 180 degrees, respectively.

### Perspiration measurement

Capacitance sensors (AramoTS of Aram HUVIS, Korea) measured the amount of moisture on the surface of the skin to estimate perspiration at the center of the palm, forehead, and philtrum. The capacitance values of the skin and the measurement probe are proportional to the dielectric constant because the moisture level of the skin is much higher than that of other substances [28]. Hence, in this study, we assumed that the electric level of other substances was negligible, and the capacitance values could be directly transformed to the skin surface moisture amount with an arbitrary unit (AU) ranging from 1 to 100. The performance of the capacitance sensor to measure moisture amounts is mainly dependent on the contact area between skin surface and electrodes. To maintain the skin contact area to be consistent and independent of the individual subjects, during the measurement, it was necessary to keep constant hold-down force in the direction normal to the skin surface. We trained the operator to perform repeated measures with the coefficient of variation within 10%.

Before the clinical study, we conducted a pilot study for the reliability of perspiration measurement in various body parts such as forehead, philtrum, chest, back, back of the hand, palm, top of the foot, and sole, etc. In this preceding experiment, we found that forehead, philtrum, and palm were the most repeatable among the candidates. It was difficult to consistently measure perspiration at the chest and back due to clothes. Some body parts such as back of the hand, top of the foot, and sole did not induce significant perspiration from our heating stimulus conditions.

Perspiration was induced by heating the back of the subject with an electrical heating system for ten minutes. The moisture amount was measured at each body site twice before and twice after the heating stimulus. In a preliminary study, we found that some subjects who were emotionally agitated or physically uneasy were shown by moisture amount over 38 AU. To exclude those temporary agitated samples from the analysis, therefore, we selected 38 AU as the cut-off value. The subjects who had a pre-stimulus moisture amount greater than the cut-off value at all three sites of the skin were excluded from the experiment.

The measurements after the stimulus obtained the perspiration characteristics of the palm (P_H_), forehead (P_F_), and philtrum (P_P_). Furthermore, the moisture differences between the forehead and palm (P_FH_), philtrum and palm (P_PH_), and philtrum and forehead (P_PF_) were calculated to analyze the relative difference in perspiration for those respective locations.

### Statistical analysis

Two sample independent t-tests were performed to obtain the mean difference of characteristics between the TE and SE groups. An analysis of covariance (ANCOVA) was conducted to evaluate the effect of SC on nine skin features measured independently by the visco-elasticity and moisture sensing devices. Because several studies reported that the physical properties of human skin are influenced by age [26,29], the age effect was used as a covariate in the ANCOVA models for each of the nine skin features. The difference between the size of the TE and the SE groups was compensated for by using estimated marginal means and pooled variances.

To classify the two SC groups, we used binary logistic regression models for analysis of the combinations of significant skin features. The performance of each model was evaluated by the area under the receiver operating characteristic (ROC) curves and estimated accuracies for each model using 10-fold cross validation. Pearson correlation coefficients between each pair of significant skin features were investigated to determine a highly correlated feature set. Principal component analysis (PCA) was conducted to generate new linear combinations of the correlated features for model parsimony and sensitivity. The level of statistical significance was set at *p* < 0.01 for all analyses. Each skin feature used in classification models and PCA was standardized with a mean of 0 and a standard deviation of 1. All statistical analyses were conducted using R (version 2.15.1).

## Results

### Subject demographic characteristics

To reduce the sensitivity of classification models, two subjects were excluded under the criteria that data values lying outside of three time of interquartile range (IQR) from the upper (75th percentile of data) and lower quartile (25th percentile of data) for each feature were considered as outliers. Therefore, a total of 109 female subjects were analyzed in this study. As shown in Table 1, TE data from 63 (57.8%) subjects and SE data from 46 (42.2%) subjects were used to analyze the relevance of skin properties. There were no significant differences in age, height, pulse, or body temperature between the TE and SE groups (all *p* > 0.01). The TE group had significantly higher values than the SE group for weight (p < 0.0001), BMI (p < 0.0001), SBP (p < 0.0001) and DBP (p < 0.01). These results were similar to those in previous studies on the Sasang constitutional diagnosis.

**Table 1:** Demographic survey results according to SC types

	**Constitutions**		
**Characteristics**	**TE (n = 63)**	**SE (n = 46)**	**p-value**
Age (year)	57.5 (5.7)	56.1 (4.8)	0.1963
Height (cm)	157.2 (5.2)	156 (4.7)	0.2229
Weight (kg)	67.3 (7.2)	50.8 (5.3)	<0.0001
BMI (kg/m^2^)	27.2 (2.6)	20.8 (1.8)	<0.0001
SBP (mmHg)	126 (16.9)	113.2 (15.2)	<0.0001
DBP (mmHg)	75.4 (9.9)	69.5 (9.1)	<0.01
Pulse (time/min)	70.8 (8.8)	68.9 (7.4)	0.237
Body temperature (°C)	36.4 (0.3)	36.4 (0.2)	0.9246

Values are represented as the mean (standard deviation), P-values for each variable are derived from the result of the independent two sample t-test. BMI: body mass index, SBP: systolic blood pressure, DBP: diastolic blood pressure.

### The relevance of skin properties with the two SC types

**Figure 1:**
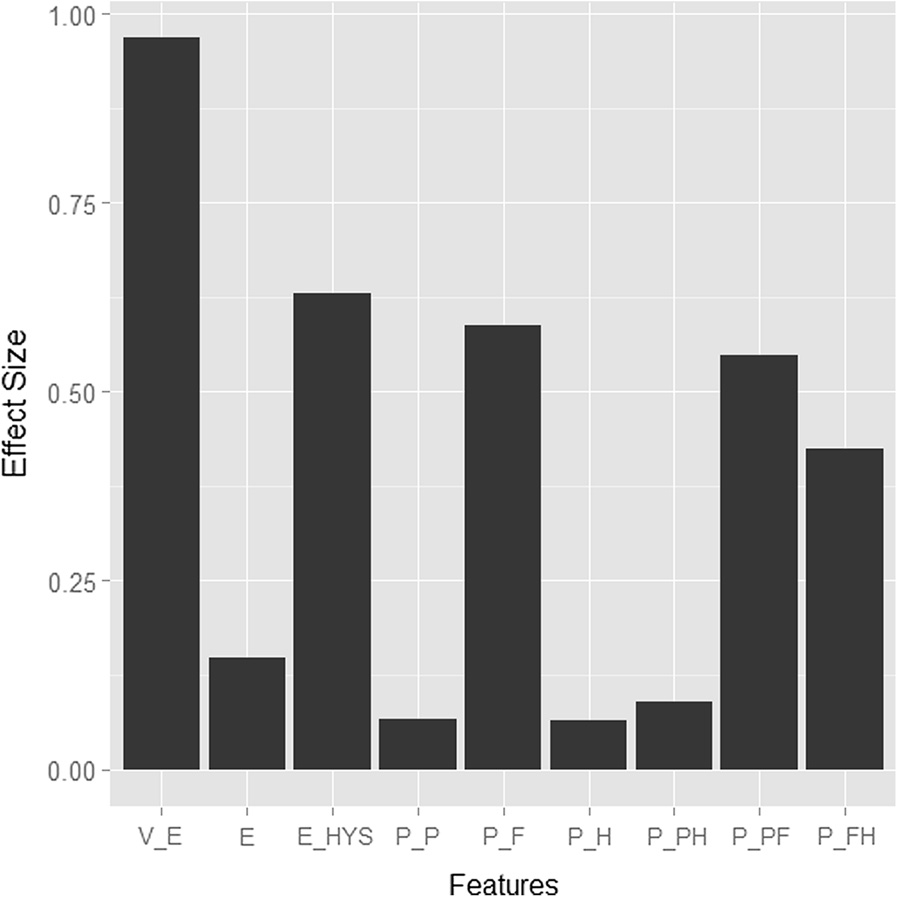
**Estimated effect sizes for nine skin features.** Each effect size (ES) was calculated using the following formula: ${\mathrm{ES}}_{\mathrm{feature}}=\left({\bar{x}}_{\mathrm{TE}}-{\bar{x}}_{\mathrm{SE}}\right)/s_{p}$, where ${\bar{x}}_{\mathrm{TE}}$, ${\bar{x}}_{\mathrm{SE}}$ represent adjusted means for the TE and SE groups, respectively, and *s*_*p*_ indicates the square root of the pooled variance of the TE and SE groups. Pooled variances for each skin feature were obtained using the mean squared error of each ANCOVA model.

The adjusted means of skin features and their standard error after adjusting for age are shown in Table 2. The effect sizes of the difference between SC groups for each skin feature are illustrated in Figure 1. For all skin features, the interaction between age and SC group (age × SC) was not statistically significant. The effect of age was not significant for all skin features except *V*_*E*_ (F(age) = 68.867, *p* < 0.001). The adjusted mean of *V*_*E*_ for the TE group was significantly higher than that of the SE group (*F* (*SC*) = 24.530, *p* < 0.001). This result indicates that the SE group shows a tendency toward slower recovery than the TE group when the skin is stretched by external forces. The ANCOVA for *E*_*HYS*_ showed a statistically significant effect for the SC group (*F*(*SC*) = 10.364, *p* < 0.01). In contrast to the results of the ANCOVA for *V*_*E*_, the TE group showed a lower adjusted mean of *E*_*HYS*_ than the SE group.

**Table 2:** Summary of ANCOVA for each measured skin variables for two constitution groups

	**Adjusted mean (S.E)**		**F (age)**	**F (age × SC)**	**F (SC)**
**Variables**	**TE**	**SE**			
*V*_*E*_	4.025 (0.087)	3.360 (0.103)	68.867***	0.002	24.530***
*E*	10.780 (0.200)	10.553 (0.236)	0.719	0.113	0.579
*E*_*HYS*_	0.135 (0.002)	0.147 (0.003)	0.105	0.724	10.364**
*P*_*P*_	42.225 (0.347)	42.409 (0.407)	0.437	0.153	0.118
*P*_*F*_	41.073 (0.262)	39.856 (0.307)	0.081	1.175	9.050**
*P*_*H*_	36.462 (0.363)	36.650 (0.426)	0.003	0.131	0.112
*P*_*PH*_	2.761 (0.621)	3.197 (0.727)	0.088	1.499	0.206
*P*_*PF*_	1.152 (0.323)	2.553 (0.378)	0.888	1.703	7.892**
*P*_*FH*_	4.611 (0.419)	3.206 (0.491)	0.049	0.984	4.710*

Values in the second and third column indicate the adjusted mean (standard error: S.E). The adjusted means for each skin feature were estimated without the interaction term in the ANCOVA models. F statistics for the effects of age and SC groups are provided in the third and the last column, respectively.

*p < 0.05, **p < 0.01, ***p < 0.001.

Regarding the results of the ANCOVA for features corresponding to perspiration including *P*_*P*_*P*_*F*_, and *P*_*F*_, only *P*_*F*_ showed a significant difference between the SC groups (*F*(*SC*) = 9.050, *p* < 0.01). The adjusted mean of the *P*_*F*_ for the TE group reports more perspiration than the SE group. The ANCOVA for relative differences of perspiration among the three body sites (*P*_*PH*_, *P*_*PF*_ and *P*_*FH*_) indicates that only the adjusted mean of *P*_*PF*_ showed a significant difference between the TE and SE groups (F(SC) = 7.892, *p* < 0.01).

### Feature dimension reduction by PCA and classification

**Figure 2:**
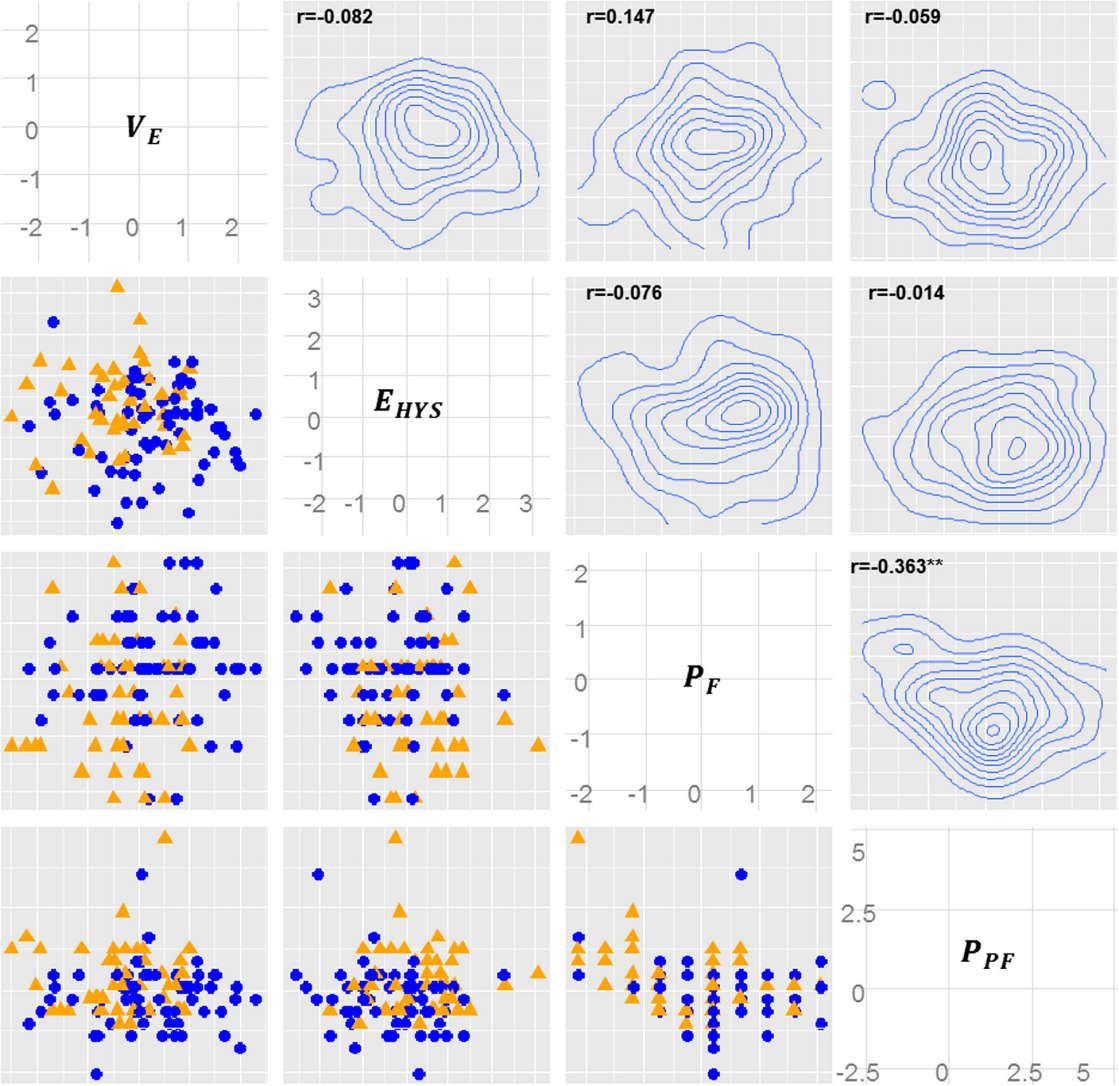
**Scatterplot matrix and density plots corresponding to all pairs of the four significant skin features.** All skin features are scaled to a mean of 0 and a standard deviation of 1. Scatterplots and density plots are illustrated in the lower triangular and upper triangular elements of the matrix plot, respectively. Blue circles and orange triangles on the scatterplots denote samples in the TE group and SE the group, respectively. Pearson correlation coefficients from the four skin features are denoted in the top left of the density plots.

Four skin features showed significant differences between the TE and SE groups, as shown by the results of the ANCOVA in The relevance of skin properties with the two SC types. Pairwise correlation coefficients and two-dimensional densities for those four skin features were examined to verify the correlation structures. These were performed to conduct a preliminary analysis for the classification of the two SC groups. As shown in Figure 2, only *P*_*F*_ and *P*_*PF*_ are significantly correlated (r = -0.363). From these correlation analysis results, the improved linear combination of both features can be considered because *P*_*PF*_ contains the information of *P*_*F*_. PCA was conducted to identify the linear combination of the two correlated features without a loss of information [30]. The first principal component (PC1) explained 68% of the total variation due to the two skin features and the loading coefficient was given by [-0.707, 0.707]. Hence, the linear combination of *P*_*F*_ and *P*_*PF*_ can be expressed as

(4) $$\mathrm{PC}1=-0.707P_{F}+0.707P_{\mathrm{PF}}=0.707\left(P_{P}-2P_{F}\right)$$

**Figure 3:**
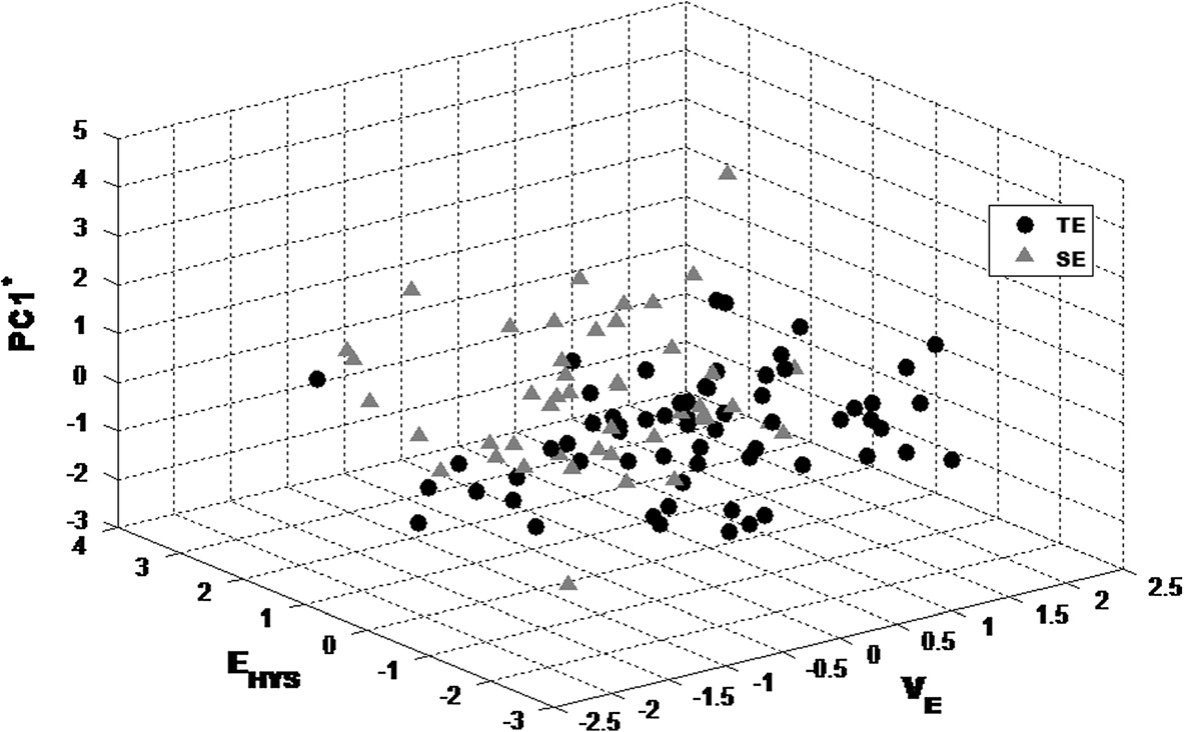
Scatter plot of the TE and SE groups in a 3 dimensional skin feature space.

In addition, a three dimensional plot of PC1 can provide a graphic understanding of the physical differences between the visco-elasticity and perspiration of the skin (Figure 3).

Binary logistic regression models with several combinations of significant skin features were applied and new features were derived from the Equation (4) to build the classification model for the two SC groups. Four models were considered: using only elasticity information (Model 1); using only perspiration information (Model 2); using both elasticity and perspiration information (Model 3); and using elasticity and PC1 (Model 4). The 10-fold cross validation results for each model are summarized in Table 3. The performance of the model was enhanced in Model 3 (AUC = 0.759 ± 0.009, accuracy = 0.695 ± 0.014) when compared with models using only one part of the information (Model 1: AUC = 0.721 ± 0.011, accuracy = 0.628 ± 0.013; Model 2: AUC = 0.674 ± 0.012, accuracy = 0.632 ± 0.013). The Model 4 (AUC = 0.764 ± 0.010, accuracy = 0.707 ± 0.012) showed slightly higher performance than that of Model 3.

**Table 3:** Area under the ROC curves and prediction accuracy based on 10-fold cross validation

**Model**	**AUC**	**Accuracy**
Model 1	0.721 (0.011)	0.628 (0.013)
Model 2	0.674 (0.012)	0.632 (0.013)
Model 3	0.759 (0.009)	0.695 (0.014)
Model 4	0.764 (0.010)	0.707 (0.012)

Values indicate the average value and standard deviation of cross validated AUC and Accuracy.

Model 1: *V*_*E*_ + *E*_*HYS*_.

Model 2:*P*_*F*_ + *P*_*PF*_.

Model 3: *V*_*E*_ + *E*_*HYS*_ + *P*_*F*_ + *P*_*PF*_.

Model 4:*V*_*E*_ + *E*_*HYS*_ + *PC*1.

AUC: area under the ROC curves.

## Discussion

Skin characteristics are known to change with health conditions and age. This means that skin characteristics reflect the health status of internal organs. SCs have been reported to be closely related to the equilibrium of internal organ functions [1]. For instance, the TE group has strong anabolic functionality and weak catabolic functionality. By contrast, the SE group has strong excretion functionality and weak digestive functionality. Varying organ functionality that accompanies different constitutions may influence the mechanisms that supply nutrition to skin tissues such as the epidermis, dermis, and subcutaneous fat layer and may also influence waste excreted by sweating. This influence of organ functionality on perspiration is described many times in Donguisusebowon as follows [6].

'It is considered that if the lesser yin person with dry cholera and obstruction and rejection had perspired at the Renzhong (philtrum) point, then he is on his way to being out of danger.’(p90)

'A lesser yin patient will definitely be cured if his yang qi ascends and induces perspiration on the Renzhong point on the first day. If his perspiration does not cease on the second or third day and he is not cured, there is no doubt that the yang cannot ascend and is exhausted. A lesser yang patient will definitely be cured if his yin qi descends and induces diarrhea, with sweat on the palms and soles on the first day.’(p145)

'The greater yin person who has coldness for six or seven days without fever and perspiration will die. If he has coldness for two of three days with fever and perspiration, the illness will be a mild case. However, if he had coldness for four of five days, with fever and little perspiration on his forehead, the illness is called the long-term infectious disease and is a serious case. This disease is caused by the exertion of the mind, which induces the Stomach duct to become asthenic, and the exterior of body cannot resist the cold and is surrounded by the cold. The situation is a battle between healthy qi and the pathogen.’(p186)

'If the greater yin person sweats a lot, he is very healthy.’(p242)

The above perspiration patterns of TE or SE represent the lung or spleen function, respectively, as these are the weakest organs for each SC. For this reason, the SC specific perspiration pattern was used importantly in the prognosis after medication.

In this study, we tried to determine the significance of these skin properties that have been emphasized by SC-based traditional medicine. The differences in mechanical features between TE and SE such as viscous behaviors (V_E_) and fatigue characteristics (E_HYS_) support the reports of Donguisusebowon [6] that state that TEs have more solid flesh and SEs have smoother flesh. These also agree with Sasangyolam [7] in stating that the skin of TEs is primarily thick and stiff, and the skin of SEs is supple and soft. Although V_E_ was regulated by aging effects, the immediate recovery capability of TEs and SEs significantly differed from each other. Furthermore, skin resistance against fatigue was higher in TEs than in SEs. From these results, it can be inferred that the skin of TEs is inherently more difficult to mechanically deform and takes shorter to recover than the skin of SEs. These mechanical properties of TE are coincident with the typical characteristics of human skin caused by the thick subcutaneous fat layer and hence, support the recent clinical study of Jang et al. [31], in which they have reported that the TE type might be significantly and independently associated with abdominal obesity and could be considered a risk factor in predicting abdominal obesity.

In conventional medicine, except in extreme cases, sweating is not considered an important symptom of disease. However, in SC medicine, the amount and location of sweating are very important in diagnosing one’s health status. These characteristics also vary according to constitution. Those with the TE constitution are judged as healthy when they sweat over the entire body, while those with the SE constitution are healthy when they sweat little across the entire body except the philtrum [32,33].

Using healthy participants, we found in this study the same results as those presented by the Sasang Constitution theories. In particular, moisture differences between the philtrum and the forehead (PC1’) delineated the relative difference between TEs and SEs regarding perspiration. By employing the mean difference of the P_F_, it was verified that TEs sweat more than SEs. Interestingly, these constitutional differences are being utilized by KMDs to predict the changes of the health status of patients after treating them by acupuncture or herbal medicine. Therefore, perspiration indicators such as P_PF_, P_F_, and PC1’ could provide objective evidence of the constitution-based health diagnosis that traditional medical doctors employ.

In addition, a combined classifier to discriminate between TE and SE groups showed better performance than an individual classifier. The 3 dimensional feature space model (Model 4) by dimension reduction provided an easier way to understand the skin diagnosis by employing a three dimensional geometrical space that kept equivalent accuracy when compared with the classifier in Model 3.

There are some limitations of our study.

We did not include TY and SY subjects in the proposed model. TY type subjects are very rare and the SCAT did not gather enough sample size to develop classification model for the SY type. On the other hand, the classification accuracy for the SY type was not sufficient for our purpose. Recently, however, the classification performance of the SCAT has been improving through a variety of cohort study. In a future research, we plan to conduct a comparative study including the SY type subjects. In particular, micro-scaled skin properties such as surface roughness, pore distribution, wrinkle shape and density are expected to be relevant feature candidates for SYs (and TYs).

In the technical aspect, the measurement reliability depends sensitively on the operator’s manipulation skill of the sensors and subjects. Since perspiration can be affected by the acute reaction of the autonomic nervous system (ANS) caused by emotional changes or physiological conditions, we excluded the subjects who were suspected to be in mental or physiological agitation states by a moisture scanner. Additional bio-feedback sensory systems such as EEG, EMG, PPG, and ECG will help monitoring the real-time ANS changes during the measurement.

## Conclusion

In summary, according to the results of our experiments, we found four significant skin features that reflect the inherent constitutional attributes of the TE and SE groups in accordance with SCM literature; the visco-elasticity, elasticity hysteresis, perspiration on the forehead and philtrum. Our findings are based on a novel interpretation of the SCM literature and will contribute to developing the constitutional health status evaluation system in SCM.

This study has yet to investigate other physical skin features such as roughness, thickness, and wrinkle shapes, which are also considered important factors that could more accurately discriminate between constitutions and determine constitutional health status. Furthermore, differences arising from aging or gender should also be studied.

## Abbreviations

SCM: Sasang constitutional medicine; SC: Sasang constitution; ANCOVA: Analysis of covariance; SE: Soeum-in; TE: Taeeum-in; TKM: Traditional Korean medicine; KMDs: Korean medical doctors; SCAT: Sasang constitutional analytical tool; ROC: Receiver operating characteristic; PCA: Principal component analysis.

## Competing interests

The authors declare that they have no competing interests.

## Authors’ contributions

YK carried out the qualitative data analysis and drafted the manuscript. BK performed the statistical analysis. CJ designed the method for the skin visco-elasticity measurement experiment and contributed to the interpretation of constitution-specific relevance. JUK designed the method for perspiration measurement and contributed to the interpretation of constitution-specific relevance. YJ participated in data collection, and contributed to the refinement of the data. KK participate in the design of the clinical trial. JK conceived of this study, was the Principal Investigator, participated in its design and coordination, and contributed to the interpretation of data and content of this manuscript. All of the authors critically contributed to the final manuscript and approved the final version.

## Pre-publication history

The pre-publication history for this paper can be accessed here:

http://www.biomedcentral.com/1472-6882/14/24/prepub
